# Evolution of intron splicing towards optimized gene expression is based on various *Cis*- and *Trans*-molecular mechanisms

**DOI:** 10.1371/journal.pbio.3000423

**Published:** 2019-08-23

**Authors:** Idan Frumkin, Ido Yofe, Raz Bar-Ziv, Yonat Gurvich, Yen-Yun Lu, Yoav Voichek, Ruth Towers, Dvir Schirman, Heike Krebber, Yitzhak Pilpel

**Affiliations:** 1 Department of Molecular Genetics, Weizmann Institute of Science, Rehovot, Israel; 2 Abteilung für Molekulare Genetik, Institut für Mikrobiologie und Genetik, Göttinger Zentrum für Molekulare Biowissenschaften (GZMB), Georg-August Universität Göttingen, Göttingen, Germany; University of Bath, UNITED KINGDOM

## Abstract

Splicing expands, reshapes, and regulates the transcriptome of eukaryotic organisms. Despite its importance, key questions remain unanswered, including the following: Can splicing evolve when organisms adapt to new challenges? How does evolution optimize inefficiency of introns’ splicing and of the splicing machinery? To explore these questions, we evolved yeast cells that were engineered to contain an inefficiently spliced intron inside a gene whose protein product was under selection for an increased expression level. We identified a combination of mutations in *Cis* (within the gene of interest) and in *Trans* (in mRNA-maturation machinery). Surprisingly, the mutations in *Cis* resided outside of known intronic functional sites and improved the intron’s splicing efficiency potentially by easing tight mRNA structures. One of these mutations hampered a protein’s domain that was not under selection, demonstrating the evolutionary flexibility of multi-domain proteins as one domain functionality was improved at the expense of the other domain. The *Trans* adaptations resided in two proteins, Npl3 and Gbp2, that bind pre-mRNAs and are central to their maturation. Interestingly, these mutations either increased or decreased the affinity of these proteins to mRNA, presumably allowing faster spliceosome recruitment or increased time before degradation of the pre-mRNAs, respectively. Altogether, our work reveals various mechanistic pathways toward optimizations of intron splicing to ultimately adapt gene expression patterns to novel demands.

## Abstract 2

An experimental evolution study involving an inefficiently spliced intron reveals that the splicing machinery, introns, and RNA quality control factors evolve in Cis and in Trans when cells optimize their transcriptome to new challenges.

## Introduction

Throughout evolution, cells acquired regulatory mechanisms to tune gene expression, which have been the subject of intensive investigations—focusing mainly on transcription and translation. Among other known mechanisms, when cells are challenged to increase protein expression levels, the DNA sequence of genes can change so as to increase transcription [1,2], support more efficient mRNA translation [3,4], or result in greater mRNA transcript stability [5,6]. Additionally, the transcription and translation machineries themselves have been shown to adapt to environmental challenges by altering the cellular pools of transcription factors [7] or tRNAs [8,9].

In evolving expression programs, adaptation often occurs either directly on the genes under pressure (“evolution in *Cis*”) [10] or indirectly, e.g., on the expression machineries, typically transcription and translation (“evolution in *Trans*”)[11,12]. These two routes of evolution are profoundly different [13], as the first (*Cis*) provides a localized solution that in principle can affect only a certain gene, while the later (*Trans*) could be the method of choice if a coordinated change in many genes is needed.

Surprisingly, although the process of splicing is central to the maturation and regulation of mRNAs in eukaryotes [14–18], its role in adapting to novel demands on gene expression has not been thoroughly investigated. During mRNA splicing, precursor mRNAs are processed to remove introns while fusing exons together to create the mature transcript. This process can provide evolutionary means to diversify the proteome towards phenotypic novelty, as the choice of intron to be excluded, as well as the exons which are found in the mature transcript, can both be regulated based on the cell’s needs [16,19,20]. An aspect of splicing evolution that has been extensively studied is gain and loss of introns, for which several molecular models have been proposed, mainly reverse transcription and recombination-mediated intron loss, intron transposition, and also exonization and intronization via mutations [21–25].

While intron loss and gain have been demonstrated experimentally [26,27], other forms of evolution through changes in splicing, such as alterations in splicing efficiency under changing conditions, have not. Adaptation of splicing efficiency is presumably essential to cellular evolution given a recent finding that splicing efficiency increases with transcription rate [28], therefore making it likely that splicing efficiency of introns is under constant selection during evolution. Yet, the mechanisms that allow this adaptation are unknown.

Here, we set to reveal whether introns or the splicing apparatus can evolve so as to alter the expression levels of genes in an adaptive manner. To this end, we engineered yeast cells to express a reporter gene, to which we inserted an inefficiently spliced intron that was fused to an antibiotic resistance gene. We then carried out a lab-evolution experiment in which cells were exposed to the drug and followed their adaptation.

Our results demonstrate that adaptations were related to splicing, and they appear to have not addressed directly the transcription or translation of the gene under selection. Two alternative adaptive routes for evolution of splicing were observed. First, we found *Cis*-acting solutions in the form of adaptive mutations that occurred in the intron itself but also, surprisingly, in an upstream exon. These mutations resulted in increased splicing efficiency and higher expression levels of the antibiotic resistance gene. We then show how one such *Cis* mutation alters the predicted RNA structure of the intron to better support splicing.

Yet, in some other evolved cells there were no mutations in *Cis*, i.e., in the gene or in its surrounding regions, but rather *Trans*-acting adaptations that have increased cellular availability of the splicing machinery. Sequencing the genomes of *Trans*-evolved colonies revealed nonsynonymous mutations in the RNA recognition motifs of two SR-like proteins that are known to have a diverse set of cellular functions related to RNA splicing and maturation. In particular, SR-like proteins were shown to support splicing by co-transcriptional recruitment of splicing factors [29–32] and were also shown to be involved in quality control of nascent mRNAs by selectively exporting from the nucleus spliced mRNAs upon completion of splicing [33,34]. Here, we show that adaptations in *Trans* that occurred through this experiment have modified the affinity of these proteins to the transcript under selection in a way that could allow its more efficient splicing.

## Results

### Low splicing efficiency of a drug resistance gene leads to stressed cells in presence of antibiotics

**Fig 1:**
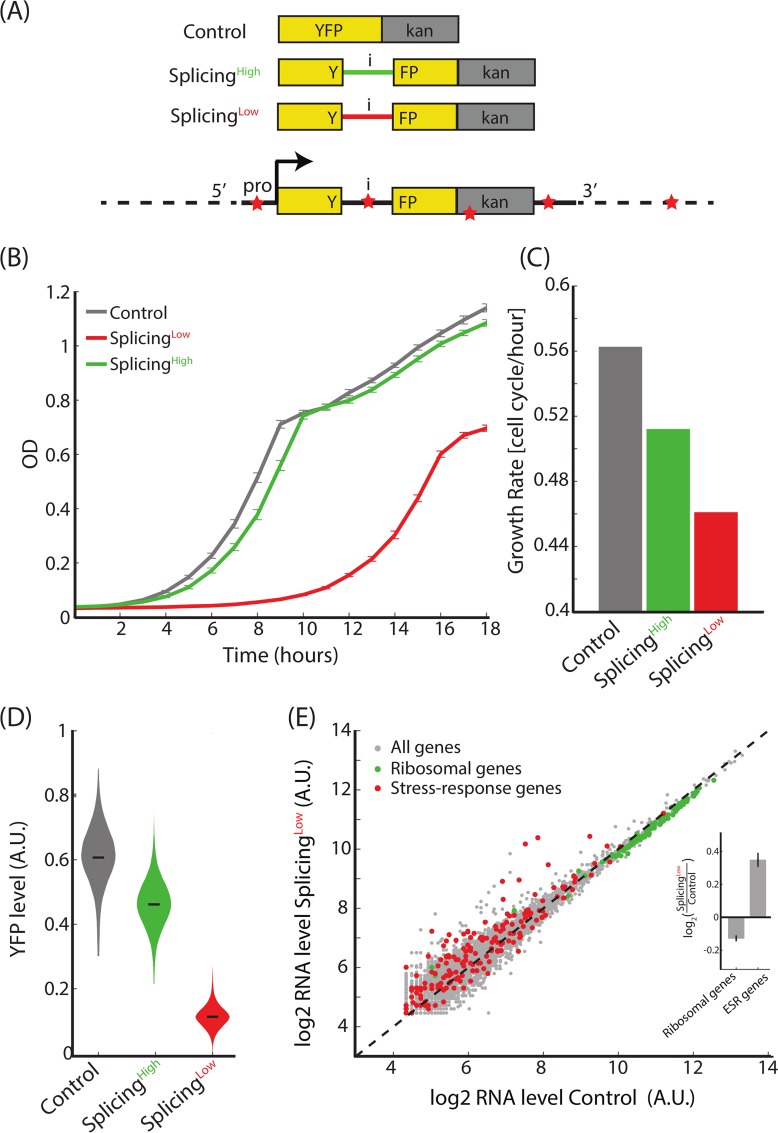
Inefficient intron splicing leads to lower gene expression levels and compromised antibiotics resistance. **(A)** We introduced two alternative introns into a YFP domain that was fused to a kanamycin resistance domain, to generate three strains: (i) Control without an intron; (ii) Splicing^High^ with an efficiently spliced intron; and (iii) Splicing^Low^ with an inefficiently spliced intron. Evolving cells at the presence of the antibiotics could adapt by mutating different parts of the YFP-Kan construct (evolution in *Cis*) or other loci, evolution in *Trans* (red stars represent potential locations of such putative mutation sites). **(B,C)** Splicing^Low^ suffers from a severe growth defect compared with Control or Splicing^High^ cells when the antibiotic is supplemented to the medium. The growth defect is manifested as both a longer lag phase and a lower maximal growth rate. **(D)** Florescence intensity of the YFP-Kan reporter for all three strains shows that Splicing^Low^ cells have lower expression levels of YFP-Kan. This observation links between YFP-Kan expression levels and cellular fitness. **(E)** Transcriptome profiling shows that ribosomal genes were down-regulated (green dots, *p* = 4.62 × 10^−26^, paired *t* test) and stress-response genes were up-regulated (red dots, *p* = 3.40 × 10^−5^, paired *t* test) in Splicing^Low^ compared with Control cells. This observation suggests that Splicing^Low^ cells experience stress because of compromised resistance to the antibiotics and that the general stress response was activated in them. (**Inset**) Mean log_2_ ratio of ribosomal and ESR gene groups. See numerical data for this figure in S1 Data.

We hypothesized that splicing efficiency of genes could serve as a means to optimize their expression levels. To test this hypothesis, we used the yeast *Saccharomyces cerevisiae*, in which approximately 30% of the transcriptome is spliced to form mature mRNAs [35] at a range of splicing efficiencies [18,36]. We built a construct that consists of two fused domains. The first is a fluorescent reporter (YFP) that was engineered to include one of two alternative natural introns—with either high or low splicing efficiency. The intron was localized near the YFP’s fluorescence site [36]. Downstream to this protein was fused an antibiotics resistance gene (kanamycin resistance gene [kan]). This general design consisted of three alternative strains: (i) “Control” with a YFP-Kan construct without an intron; (ii) “Splicing^High^” with a YFP-Kan gene that harbors the natural intron of *OSH7* that was previously reported to have high splicing efficiency within this YFP context [36]; and (iii) “Splicing^Low^” with a YFP-Kan gene that harbors the natural intron of *RPS26B*, with a low splicing efficiency [36] (see Fig 1A and S1 Table for a list of strains used in this study).

We first hypothesized that cellular growth of each strain in the presence of the antibiotic G418 will depend on YFP-Kan expression levels. We followed the growth of the three strains in the presence of the antibiotics and found that Control cells had the highest fitness, Splicing^High^ grew slower, and Splicing^Low^ demonstrated a severe growth defect compared with the two other strains (Fig 1B and 1C). We also measured fluorescence intensity of the YFP-Kan reporter in the presence of the drug and observed that Control cells demonstrated the highest fluorescence levels, followed by Splicing^High^, and with Splicing^Low^ cells showing the lowest YFP-Kan levels (Fig 1D). These results demonstrate that the inefficiently spliced intron in Splicing^Low^ reduces cellular levels of YFP-Kan and hence, presumably, leads to a reduced fitness.

Because YFP-Kan expression levels in Splicing^Low^ were significantly lower compared with the other strains, we hypothesized that Splicing^Low^ cells did not reach the needed concentration of the resistance protein to sufficiently neutralize the antibiotics, and hence resulted in stressed cells. To test this hypothesis, we performed mRNA sequencing of exponentially growing Control and Splicing^Low^ cells in an antibiotics-containing medium and analyzed the transcriptome profiles of these cells. Indeed, we observed that ribosomal genes were down-regulated in Splicing^Low^ compared with Control cells—a clear signature of stressed cells [37] (Fig 1E). Notably, we observed an averaged 8% reduction for mRNA levels of ribosomal proteins between Control and Splicing^Low^ cells and, correspondingly, an 18% reduction in growth rate. Interestingly, this is consistent with the correlation observed in a recent study between growth rate and ribosomal expression levels in yeast cells [38]. In parallel, stress-related genes [39] were up-regulated in Splicing^Low^ cells compared with Control cells (Fig 1E). We thus concluded that the general stress response was activated in Splicing^Low^ cells.

### Rapid evolutionary adaptation increases expression level of the resistance gene

**Fig 2:**
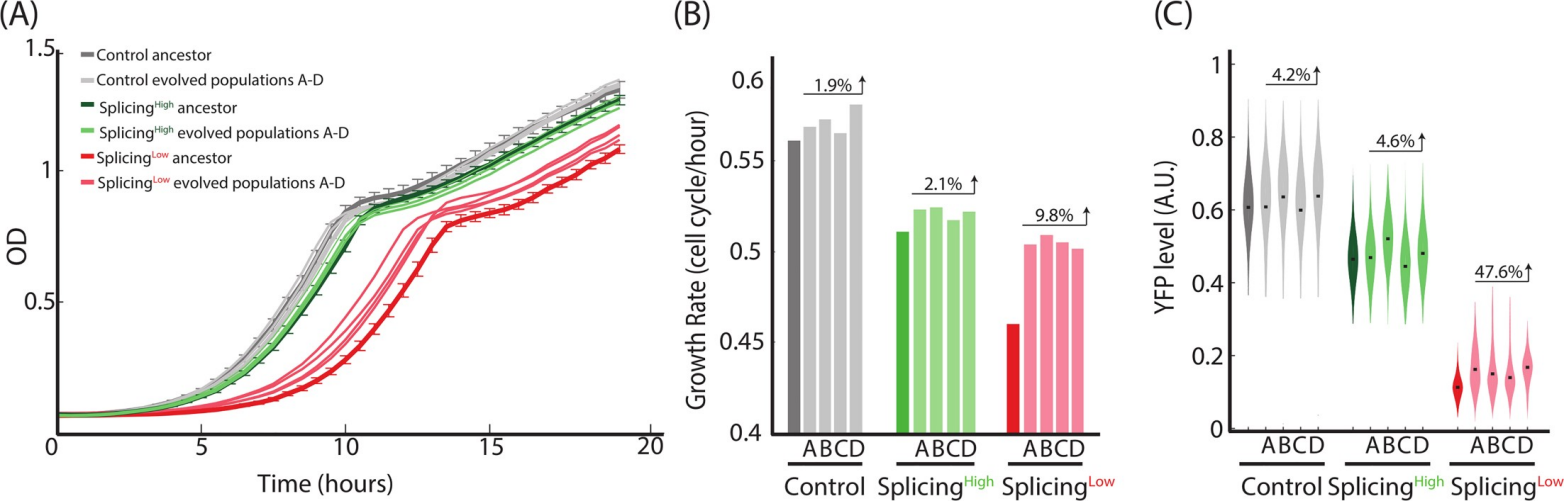
Rapid adaptation to the presence of the antibiotics is observed only for Splicing^Low^ cells. **(A,B)** We evolved Control, Splicing^High^, and Splicing^Low^ cells for approximately 560 generations with the presence of the antibiotics in four independent cultures for each strain. Growth measurements of evolved populations compared with the three ancestors shows that only evolved Splicing^Low^ cells demonstrate significant improvement in growth for all four independent evolution lines. The number above each group of evolved populations represents the average improvement in growth rate compared with these populations’ ancestor strains. These observations suggest that the inefficiently spliced intron led to a rapid adaptation of Splicing^Low^ cells. **(C)** Fluorescence intensities of the YFP-Kan reporter for all evolved cultures show that expression levels were much increased in all four evolved cultures of Splicing^Low^ compared with the ancestral strain (effect sizes = 78.67, 79.54, 75.17, 83.19). Conversely, the increase in expression levels in the evolved Control and Splicing^High^ populations were smaller (Control effect sizes = 64.66, 68.44, 63.51, 67.74; Splicing^High^ effect sizes = 54.33, 70.66, 52.43, and 58.27). The number above each group of evolved populations represents the average increase in YFP-Kan levels compared with these populations’ ancestor strains. These observations suggest that adaptation of Splicing^Low^ cells was based on their ability to increase expression levels of the resistance proteins. See numerical data for this figure in S1 Data.

Our experimental system mimics an evolutionary scenario in which there is an immediate and continuous selection pressure to up-regulate the expression level of a specific gene in a particular environment. How would the system evolve to better resist the antibiotics? Possible means to adapt include mutations in the gene’s promoter to increase transcription, mutations that increase translation initiation or efficiency, or mutations inside the gene itself that could increase the specific activity of the protein (Fig 1A). Additionally, the splicing machinery may also take part in adaptation of gene expression levels. To find which evolutionary tracks are used by cells as they adapt, we evolved the three strains by daily serial dilution on a medium supplemented with G418 for approximately 560 generations, in four independent cultures for each strain. Interestingly, only the cultures of Splicing^Low^ cells demonstrated a significant improvement in fitness when grown under the drug at the end of the experiment (Fig 2A and 2B). This observation suggests that only Splicing^Low^ experienced a sufficiently strong selective pressure to adapt to the presence of the antibiotics in the medium, in contrast to the Control and Splicing^High^ strains, which originally had much higher levels of the resistance proteins.

Consistent with the fitness measurements, YFP measurements of the evolved cultures showed that expression levels of the YFP-Kan fusion gene increased in all four evolved cultures of Splicing^Low^ compared with the ancestral strain (Fig 2C). Conversely, the increase in YFP-Kan expression levels in the evolved Control and Splicing^High^ populations was significantly smaller (Fig 2C). These results further indicate that Splicing^Low^ cells experienced the strongest selective pressure to adapt rapidly to the presence of the antibiotics, and that they achieved this goal by increasing the levels of the YFP-Kan reporter. We next moved to reveal the molecular mechanisms underlying this evolutionary adaptation.

### Adaptations in both *Cis* and *Trans* lead to increased splicing efficiency

**Fig 3:**
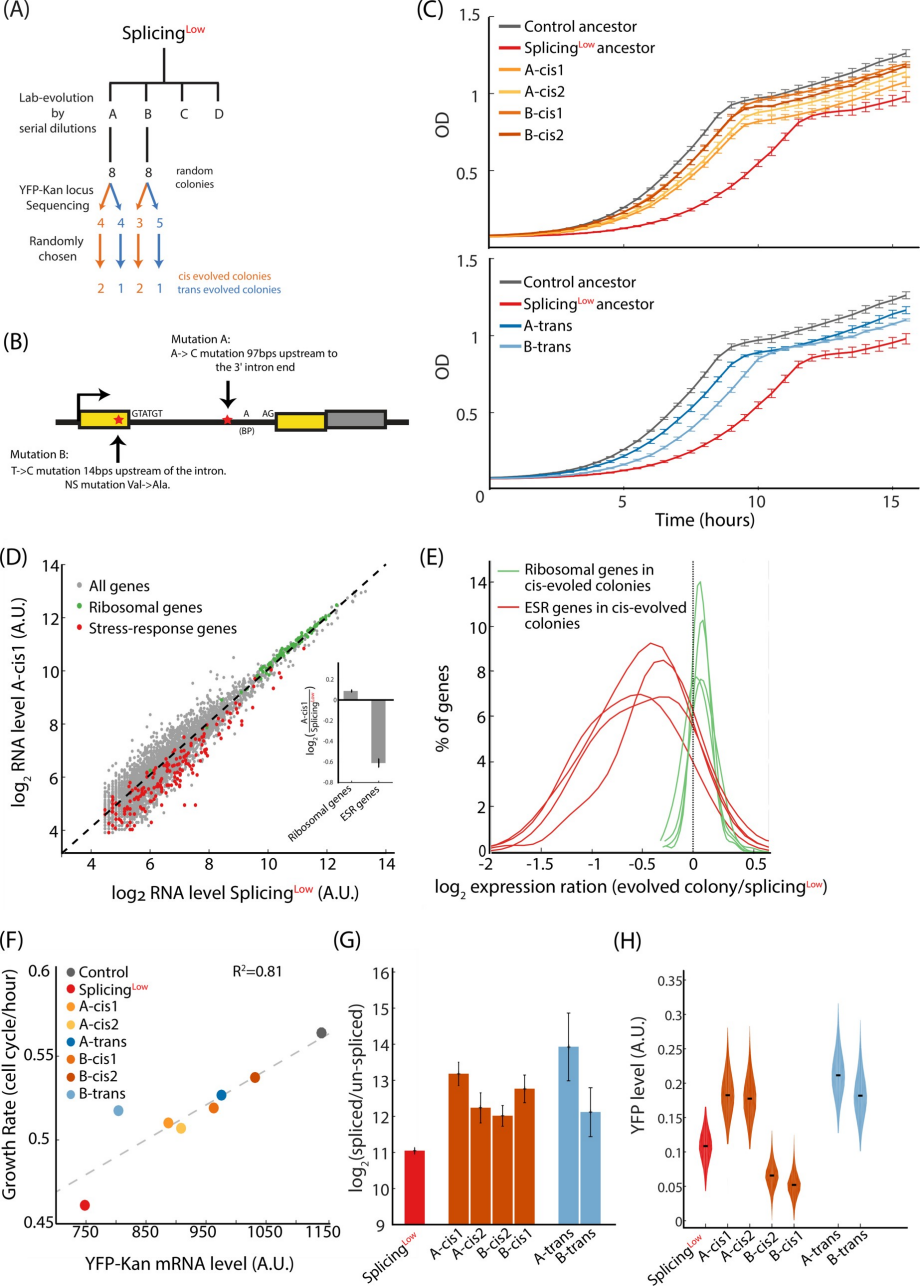
Evolved colonies demonstrate increased splicing efficiency that results in higher transcript levels and relieved stress. **(A)** We randomly chose 16 colonies in total from two evolved lines of Splicing^Low^ and sequenced the YFP-Kan locus of those colonies. We found that approximately half had mutations in the YFP-Kan construct (indication of evolution in *Cis*) and the other half did not (indication of evolution in *Trans*). Of those colonies, we randomly chose two *Cis*-evolved and one *Trans*-evolved colonies from each evolved population for further examination. **(B)** Sequencing of the YFP-Kan construct in the evolved colonies revealed two mutation types: (i) in the intron itself and (ii) in the upstream exon. These mutations did not occur in the intron 5′ donor, 3′ acceptor, or the branching point—suggesting that other positions of the intron and its vicinity are functional and may affect splicing. **(C)** All *Cis*-evolved colonies (upper graph) and *Trans*-evolved colonies (lower graph) show increased fitness compared with the Splicing^Low^ ancestor, yet still lower than the Control ancestor. **(D)** Transcriptome profiling reveals that ribosomal genes were up-regulated (green dots, *p* = 4.94 × 10^−18^, paired *t* test) and stress-related genes were down-regulated (red dots, *p* = 3.64 × 10^−15^, paired *t* test) in the evolved colony A-cis1 compared with the Splicing^Low^ ancestor. **(Inset)** Mean log_2_ ratio of ribosomal and ESR gene groups. **(E)** The four *Cis*-evolved colonies show similar trends, i.e., increased expression levels of ribosomal genes and decreased expression levels of stress-response genes (*p*-values for all cases < 0.005, paired *t* test). These observations suggest that the stress experienced by the evolved colonies was alleviated during their adaptation to the antibiotics in the medium. **(F)** mRNA levels of YFP-Kan transcripts correlate with growth rates (R^2^ = 0.82, *p* = 0.0023), suggesting that cellular fitness in our setup is indeed determined by the availability of kanamycin resistance proteins to overcome the antibiotics. **(G)** All *Cis*- and *Trans*-evolved colonies demonstrate increased splicing efficiency of the YFP-Kan mRNA compared with the Splicing^Low^ ancestor (*p* < 0.05 for all colonies compared with Splicing^Low^ ancestor). This result suggests that all adaptation trajectories led to the adaptation of the splicing process to better mature the un-spliced YFP-Kan transcript. **(H)** Fluorescence intensity of the YFP-Kan reporter shows increased levels for the two *Cis*-evolved colonies with the mutation in the intron and for the two *Trans*-evolved colonies. In contrast, the two *Cis*-evolved colonies with the nonsynonymous mutation in the exon demonstrate decreased YFP-Kan levels. This observation suggests that the nonsynonymous mutation hampered the ability of the YFP domain to fluorescent and reduced the fluorescence intensity per protein molecule (see text for full explanation). See numerical data for this figure in S1 Data.

We hypothesized that improving the low splicing efficiency of the intron in Splicing^Low^ could be natural selection’s means to adapt towards increasing the resistance gene expression levels. We therefore sequenced the YFP-Kan locus, covering the entire gene from promoter to terminator, in 16 colonies from two evolved populations (termed here population A and population B) of Splicing^Low^. Interestingly, we found that the colonies were split into two types—either with or without a mutation in the YFP-Kan locus (Fig 3A). In population A, we found that the same mutation occurred in four out of eight colonies, changing adenine to cytosine inside the intron, 97 nucleotides upstream to its 3′ end (Fig 3B). In population B, we identified an exonic nonsynonymous mutation that changed a thymine to cytosine 14 nucleotides upstream of the intron (V61A change in the YFP protein) in three out of eight colonies. In five other colonies from this population there were no mutations in the YFP-Kan locus.

Notably, none of the colonies demonstrated a mutation in the construct’s promoter, terminator, or in the sequence of the Kan resistance gene itself. These results propose that different mutations in the intron, or its vicinity, were adaptive and might affect splicing efficiency of the intron. Surprisingly, the observed mutations did not occur in the 5′ donor, 3′ acceptor, nor in the intron branch point—suggesting that other positions of the intron can also be functional by affecting splicing efficiency.

While the intron- and exon-mutated colonies represent an evolutionary adaptation in *Cis*, the colonies that showed no mutation in the entire gene construct, that coexist with the *Cis*-evolved colonies in the same populations, potentially found adaptive solutions in *Trans* that may have occurred elsewhere in the genome. We thus randomly chose six colonies: four colonies with a *Cis* mutation and two colonies that showed no mutations in *Cis*, as we reasoned that such colonies may have adapted in *Trans*. We termed these colonies according to the evolution lines from which they were derived: A-cis1, A-cis2, B-cis1, B-cis2, A-trans, and B-trans. We followed the growth of these evolved colonies in the presence of G418 and found, as expected, that all grew faster than the Splicing^Low^ ancestor (Fig 3C).

We then performed mRNA sequencing and transcriptome analyses of all colonies and found that they indeed demonstrate relaxation of the stress state that was featured in the ancestor. In particular, the general stress response genes were reduced relative to their high levels in the Splicing^Low^ ancestor, and ribosomal proteins were up-regulated relative to their low levels in this ancestral strain (Fig 3D for colony A-cis1). These dynamics were shared by all the *Cis*-evolved colonies (Fig 3E), demonstrating the robustness of this observation. Specifically, in all four *Cis*-evolved colonies, the expression levels of most ribosomal genes were increased compared with the Splicing^Low^ ancestor (percentage of up-regulated ribosomal genes in the four colonies, 86%, 63%, 83%, and 84%) and levels of the ESR genes were mostly reduced compared with the ancestor (percentage of down-regulated ESR genes in four colonies, 90%, 76%, 87%, and 84%). These findings suggest that indeed these colonies adapted to the presence of the antibiotics in the environment and that the stress experienced by them was partially alleviated.

We next hypothesized that cellular fitness might correlate with mRNA levels of the YFP-Kan construct because increased transcript levels should result in higher concentrations of the YFP-Kan protein. Indeed, maximal growth rates of the Control and Splicing^Low^ ancestors and of the six evolved colonies correlate with mRNA levels of their YFP-Kan construct, as deduced from the RNA-seq (Fig 3F).

Because the observed *Cis* mutations occurred at the vicinity of the intron, we hypothesized that they increased splicing efficiency of the YFP-Kan transcript. To test this possibility, we assayed splicing efficiency for both *Cis*- and *Trans*-evolved colonies with qPCR, targeting the un-spliced or spliced transcript versions. Interestingly, the ratio of spliced to un-spliced transcripts, a measure of splicing efficiency, was higher in all evolved colonies compared with the Splicing^Low^ ancestor, suggesting that at least some of the increase in mRNA level we observed in the evolved colonies results from higher splicing efficiency (Fig 3G).

To prove that adaptation of the colonies actually led to higher protein levels of the fluorescence-resistance fused protein, we measured fluorescence intensity using flow cytometry. We found that the two Cis-evolved colonies from population A (A-cis1 and A-cis2) and the two *Trans*-evolved colonies (A-trans and B-trans) showed higher YFP-Kan levels compared with the ancestor. In contrast, the two *Cis*-evolved colonies from population B (B-cis1 and B-cis2) demonstrated decreased fluorescence intensity values relative to the ancestor (Fig 3H). These observations indicate that the nonsynonymous, exon mutation reduced the per-protein fluorescence value of the YFP component of the YFP-Kan construct in these colonies. Indeed, this exonic mutation occurred in a position that was recently reported to reduce fluorescence when mutated in the highly similar GFP [40]. Because YFP’s functionality, i.e., fluorescence, was not selected for or against in our setup, it appears to have been free to obtain mutations that help achieve a higher expression level of the entire fusion construct. It thus seems that a modular domain architecture of a protein may increase its evolvability under relevant conditions, as it allows the trade-off and optimization of one domain at the expense of another.

**Fig 4:**
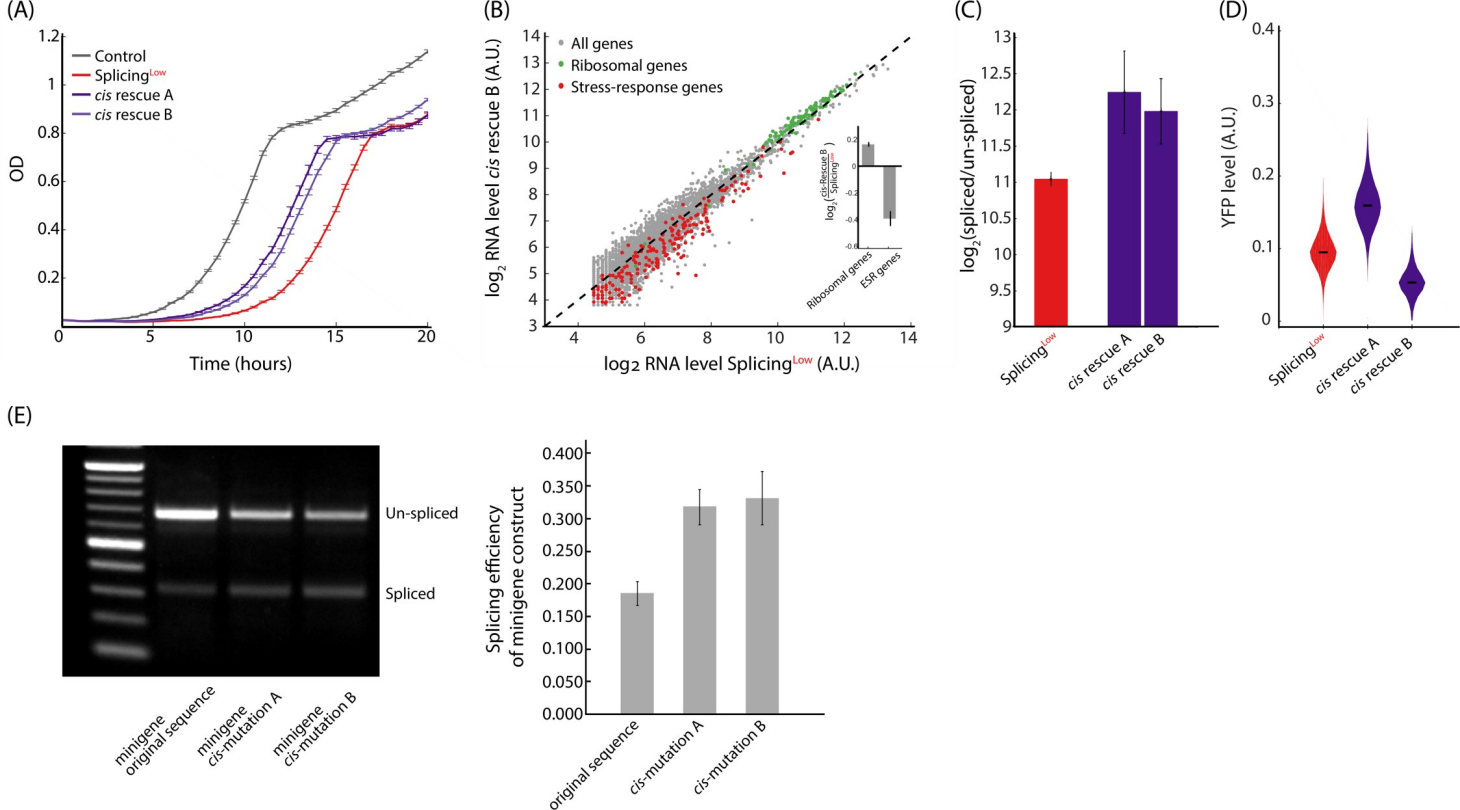
*Cis*-acting mutations are sufficient to increase fitness by elevating splicing efficiency. **(A)** We created two *Cis*-rescue strains, each harboring one of the mutations that appeared spontaneously in the evolved populations. Growth of the two *Cis*-rescue strains show that a single mutation in the YFP-Kan construct is sufficient to increase fitness compared with Splicing^Low^. **(B)** The exonic mutation is also sufficient to alleviate stress, as ribosomal genes were up-regulated (green dots, *p* = 1.02 × 10^−18^, paired *t* test) and stress-related genes were down-regulated (red dots, *p* = 9.02 × 10^−12^, paired *t* test) in *cis*-Rescue-B compared with Splicing^Low^. The same trend was also observed for the intronic mutation for *cis*-Rescue-A cells. (**Inset**) Mean log_2_ ratio of ribosomal and ESR gene groups. **(C)** The two *Cis*-rescue strains demonstrate higher splicing efficiency of the YFP-Kan mRNA compared with the Splicing^Low^ ancestor (*p* < 0.05). This result suggests that a single mutation is sufficient to improve splicing efficiency. **(D)** Fluorescence intensity of the YFP-Kan reporter for the *cis*-Rescue-A and *cis*-Rescue-B strains show similar trends as the colonies in Fig 3D—supporting earlier conclusions. **(E)** The effects of *Cis* mutations on splicing tested with a mini-gene approach. We cloned the intron’s original sequence and its two mutated versions together with 200 bps surrounding it into a high–copy number plasmid. RT-PCR assays for WT (BY4741) cells transformed with these plasmids show that, even in this context, the *Cis* mutations are sufficient to increase splicing efficiency of the intron for both mutated intron versions compared with the original sequence (*p* < 0.05, *t* test). See numerical data for this figure in S1 Data. WT, wild-type.

We next wanted to confirm that the fitness gained in the *Cis*-evolved colonies was indeed due to these mutations. It is still possible that additional beneficial mutations exist in the genome of the *Cis*-evolved colonies, which account for at least part of the improved fitness we observed in these colonies. We thus generated two rescue strains, termed *cis*-Rescue-A and *cis*-Rescue-B, in which these *Cis*-acting mutations were introduced individually to the ancestral Splicing^Low^ background. Notably, the two rescue strains grew better than Splicing^Low^ cells in the presence of the antibiotics, although not as well as Control cells (Fig 4A). Additionally, the stress experienced by Splicing^Low^ cells, as observed by changes in expression levels of ribosomal and stress genes, was alleviated upon insertion of each individual *Cis* mutation (Fig 4B). Then, we measured splicing efficiencies and fluorescence intensity levels for both rescue strains and found that they resembled the levels shown in the evolved single colonies (Fig 4C and 4D, in comparison with Fig 3G and 3H).

Our data so far demonstrate that the *Cis* mutations we identified in our lab-evolution experiment are beneficial because they improve splicing efficiency in the context of the entire YFP-Kan transcript. To further demonstrate that indeed these mutations increase splicing efficiency and to better exclude other mechanisms that might affect the YFP-Kan transcript, we used a mini-gene approach, a widely used methodology in splicing studies [41]. In this approach, the intron together with its adjacent exons are cloned to a new context, and splicing efficiency is measured. To this end, we cloned the original sequence of the intron plus 200 bases up- and downstream of it into a 2μ plasmid to a locus driven by a strong promoter. We additionally created two mutated versions of this construct, each with one of the two *Cis* mutations we discovered. Because there are only two short exons surrounding the intron in this system, it is ideal to explore the effects of the mutations on splicing. We thus harvested mRNA from exponentially growing cells and measured the splicing efficiency of each variant using PCR, with primers flanking the intron that simultaneously amplify both spliced and un-spliced versions of the YFP-Kan transcripts. In agreement with the splicing assay done for the full gene (Fig 4C), in the mini-gene assay too, both variants with either mutation *cis* A or B showed increased splicing efficiency compared with the ancestral versions (Fig 4E).

Our results thus far provide direct evidence that intron splicing takes part in the adaptation and optimization of gene expression patterns to environmental needs. Although intron sequences are much less conserved, compared with exons, and are believed to be less functional, we demonstrate that their sequence can be used by natural selection as a molecular mechanism to regulate splicing efficiency and adjust gene expression patterns.

### *Cis* mutation is adaptive through effects on mRNA structure that make 5′ donor and branching point sites more accessible to splicing

**Fig 5:**
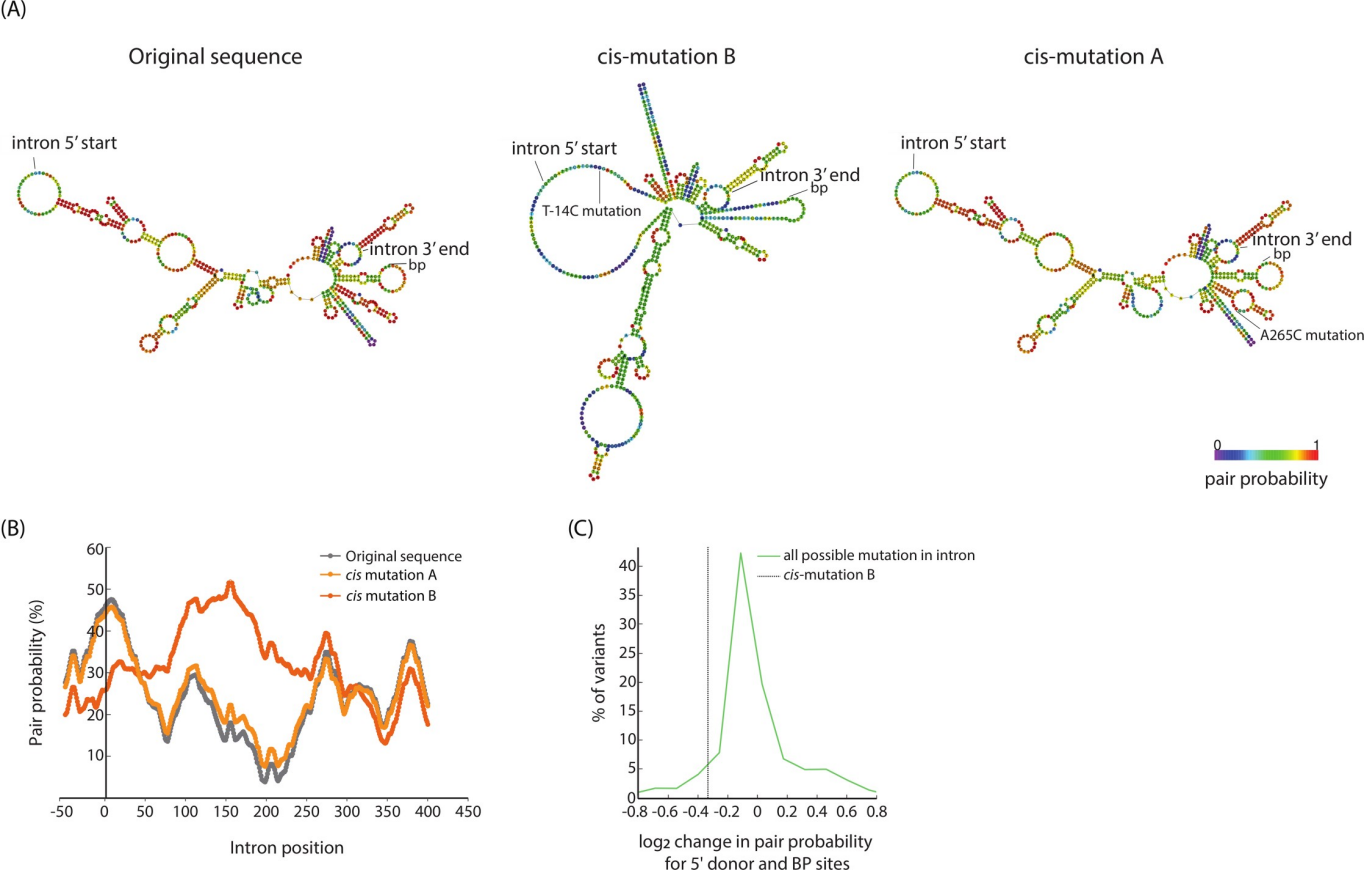
A secondary structure model of the effects of *Cis* mutation on splicing efficiency. **(A)** RNA secondary structure predictions using Vienna algorithm of the intron plus 50 bps of its surrounding exons for the original sequence and its two mutated versions. *Cis*-mutation B reshapes the predicted secondary structure and lowers the base pairing probability around the 5′ donor and branching point sites. **(B)** Base pairing probability at all positions as calculated by the Vienna algorithm for the original intron sequence and its two mutated versions. Position of 5′ donor site is 0, position of branching point site is 328, position of 3′ acceptor site is 361, position of mutation A is 265, position of mutation B is −14. **(C)** Probability distribution of the log_2_ of change in base pairing probability along a 20-base window at the 5′ donor and branching point sites for all possible single nucleotide variations on the intron's sequence compared with the original sequence. Vertical line denotes the value of the observed *cis*-mutation B, which is at the bottom 5.5 percentage of the histogram; i.e., it is among mutations that mostly reduce the base pair probability at these sites.

How can the mutations we identified in the intron facilitate splicing? One possibility is that these changes favorably alter the RNA structure of the un-spliced YFP-Kan transcript by making the splicing sites more accessible to the spliceosome. Indeed, RNA structures can inhibit or facilitate binding of spliceosome components to the pre-mRNA and affect splicing efficiency [42,43]. We thus computationally modeled the RNA structure of the intron and 50 bases on both its sides using the ViennaRNA algorithm [44]. We performed this analysis for the original and the two mutated sequences of the intron. Interestingly, mutation *cis* B, located near the 5′ donor site, leads to massive changes in the predicted RNA structure, notably causing the structure near the 5′ donor and the branching point sites to loosen (Fig 5A). Specifically, the pairing probability, a prediction for how likely it is for a position along the RNA molecule to associate with other positions, is decreased at the 5′ donor and branching point positions between the original and the mutated sequence (Fig 5B). How likely is a single point mutation to change so drastically the predicted structure of an RNA? To ask that, we constructed a simple null model in which we calculated the predicted pair-probability difference between the original sequence and each of the other possible single nucleotide mutations in the intron. Notably, mutation *cis* B falls among the 5.5% of all mutations with the highest predicted potential to affect the secondary structure; i.e., it is among the 5.5% of point mutations that loosen the RNA secondary structure near the 5′ donor and branching point the most (Fig 5C). These observations suggest a model in which mutation *cis* B may facilitate splicing due to increased accessibility of the splicing machinery to the functional splicing sites. In contrast, mutation *cis* A did not show similar patterns to mutation *cis* B (Fig 5A and 5B), raising the possibility that a different, still obscure, mechanism is causing the beneficial effects of this mutation.

### Evolution in *Trans*: Increasing cellular availability of the splicing machinery can be adaptive

We next aimed to decipher the mechanism behind the increased YFP-Kan levels in the *Trans*-evolved colonies that showed no mutations in *Cis*, i.e., within the reporter gene or in its vicinity. We reasoned that elevating availability of the splicing machinery could be a means to increase splicing efficiency of the YFP-Kan transcript and thus could be used as an adaptive mechanism to the antibiotics challenge. As with other cellular machineries whose functioning depends on supply-to-demand economy [4,8,45–47], increased splicing availability could be achieved by either increasing the expression of the splicing machinery genes or decreasing expression levels of other intron-containing genes, which collectively constitute the “demand” for the splicing machinery.

**Fig 6:**
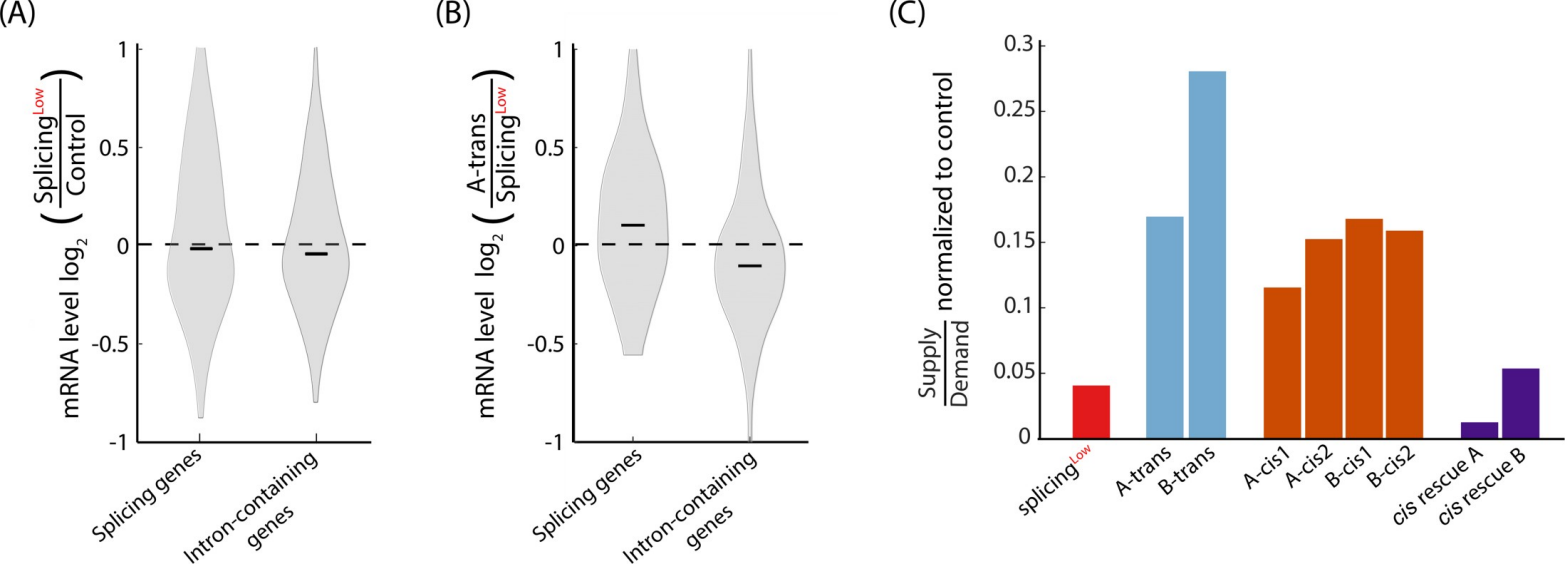
Increasing cellular availability of the splicing machinery is an adaptive mechanism of splicing. **(A)** The groups of splicing genes (splicing supply) and intron-containing genes (splicing demand) show similar levels between Splicing^Low^ and Control ancestors (*p* > 0.05, paired *t* test for both gene groups). **(B)** The groups of splicing genes and intron-containing genes were increased (*p* = 1.36 × 10^−3^, paired *t* test) and decreased (*p* = 1.67 × 10^−2^, paired *t* test), respectively, in colony A-*trans* compared with the Splicing^Low^ ancestor. This observation suggests that the supply-to-demand ratio of the splicing machinery was increased in the A-*trans* colony, which allowed its increased splicing efficiency of the YFP-Kan transcript. **(C)** Supply-to-demand ratios for the splicing machinery were calculated to Control and Splicing^Low^ ancestors, to all evolved colonies, and to the *Cis*-rescue strains as the sum expression level of all splicing-related genes over the sum expression level intron-containing genes. Values of all strains were then normalized to the value of the Control ancestor. Importantly, supply-to-demand ratios are similar for all strains that did not evolve (Control and Splicing^Low^ ancestors and the two rescue strains) and were increased for all evolved colonies (*p* = 0.005 for difference in supply-to-demand ratios between evolved and non-evolved strains, *t* test). These results suggest that indeed the cellular availability of the splicing machinery was elevated due to an evolutionary adaptation process and not because of other physiological mechanisms (see Discussion). See numerical data for this figure in S1 Data.

To test if any of these evolutionary routes were indeed taken by the evolved cells, we calculated the expression level ratio of genes between the evolved colonies and their Splicing^Low^ ancestor. In colony A-*trans*, we observed increased expression ratio of splicing machinery genes (the “supply”) and decreased expression ratio of non-ribosomal intron-containing genes (the “demand,” Fig 6B, and S1 Fig for other colonies). In contrast, both supply and demand genes show similar expression levels between Splicing^Low^ and Control ancestors, suggesting that a mere physiological adaptation did not confer upon the Splicing^Low^ ancestor the ability to adjust supply and demand to the intron burden (Fig 6A and see Discussion).

We then computed changes in “supply-to-demand splicing availability” by summing up for each strain the expression levels of the splicing machinery genes and intron-containing genes, separately, then dividing these values and normalizing the ratio with that of the control strain. Interestingly, the supply-to-demand difference has increased appreciably in the *Trans*-evolved colonies compared with the Splicing^Low^ ancestor. The *Cis*-evolving colonies have also improved their supply-to-demand relative to the ancestor, indicating that they too may have evolved in *Trans* in addition to their *Cis* adaptations. Thus, we concluded that both *Cis* and *Trans* adaptation routes can co-occur in the same genome towards optimization of its gene expression patterns. Notably, the group of non-evolved strains (Splicing^Low^ and the two *Cis*-rescue strains) showed lower supply-to-demand ratios compared with the group of evolved strains (both *Cis*- and *Trans*-evolved colonies) (Fig 6C).

Taken together, we argue that increased cellular availability of the splicing machinery is the result of an evolutionarily adaptive process, which might have allowed for the improved splicing efficiency of the YFP-Kan gene in the evolved colonies. While genomic sequencing revealed interesting mutations in *Trans* (see below), we did not find mutations that can explain these supply-to-demand changes in a straightforward way.

### Adaptation in *Trans* is also based on mutations in SR-like proteins

**Fig 7:**
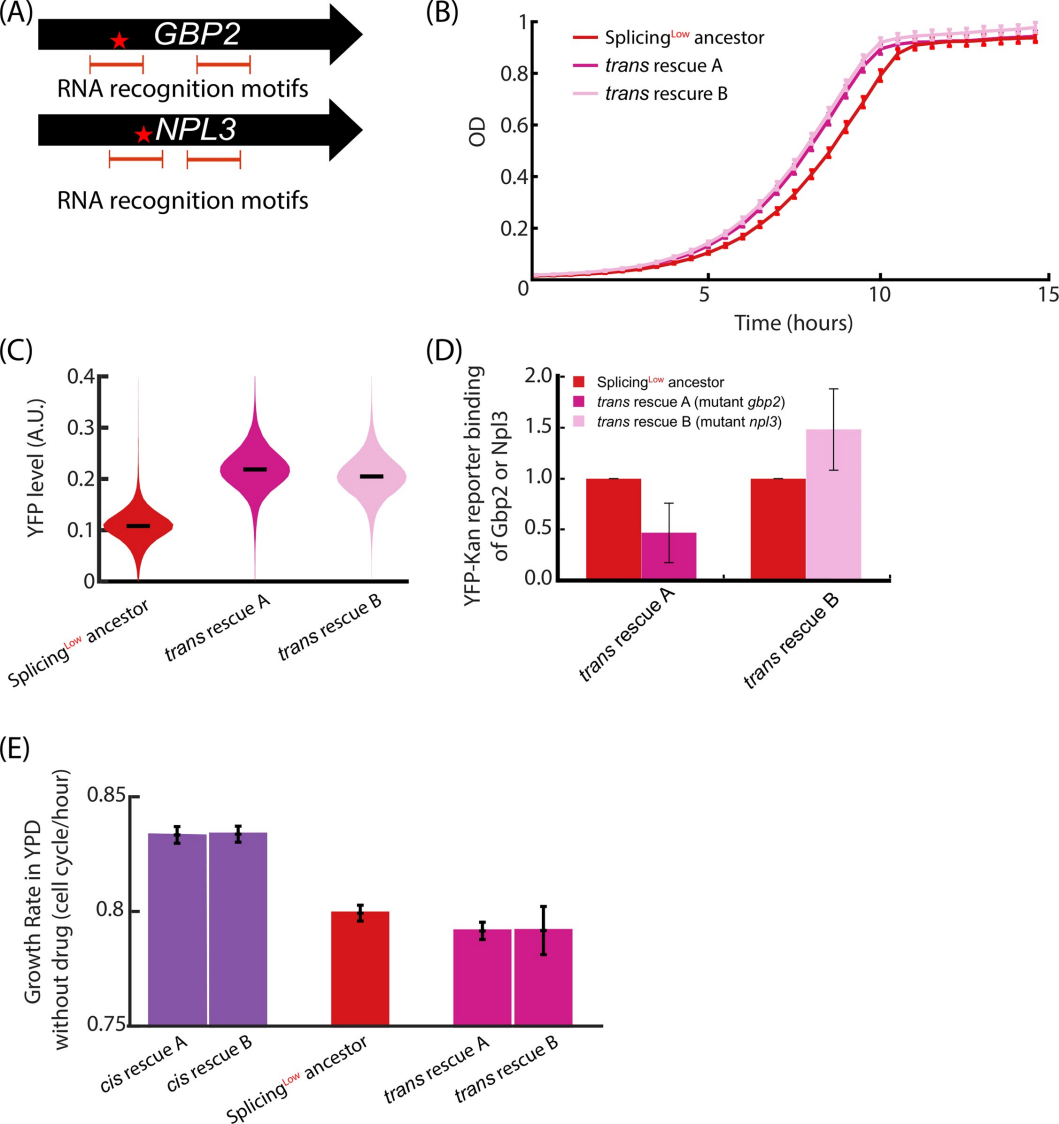
*Trans* adaptation is based on mutations in SR-like proteins that change their affinity to the YFP-Kan transcript. **(A)** Genome sequencing of the *Trans*-evolved colonies revealed two mutations in the SR-like proteins *GBP2* and *NPL3*. Both nonsynonymous mutations, H160Y in *GBP2* and F160V in *NPL3*, occurred in an RNA recognition motif. **(B)** We created two *Trans*-rescue strains, termed *trans*-Rescue-A and *trans*-Rescue-B, each harboring one of the two mutations as described in A. Growth of these two *Trans*-rescue strains in the presence of the drug show that the *Trans* mutations are sufficient to increase fitness compared with Splicing^Low^. **(C)** Fluorescence intensities of the YFP-Kan reporter for *trans*-Rescue-A and *trans*-Rescue-B strains are higher compared with Splicing^Low^ cells. **(D)** Binding of mutant gbp2 and npl3 to the YFP-Kan mRNA was examined and compared with the respective WT proteins. RT-PCR results showed that mutant gbp2 has a reduced binding affinity to the reporter (*p* = 2.97 × 10^−3^), whereas mutant npl3 binds with a slightly higher affinity (*p* = 9.54 × 10^−2^). These opposite effects on binding can be rationalized by the different functions of *GBP2* and *NPL3*. See text for full details. **(E)** Growth rates of all four rescue strains and the Splicing^Low^ ancestor under permissive conditions, without the drug. Notably, *Cis*-rescue strains demonstrate higher growth rates compared with Splicing^Low^—indicating that they alleviate the burden of the inefficiently spliced intron that is independent of the drug. In contrast, both *Trans*-rescue strains show similar growth rates compared with Splicing^Low^—indicating that mutations in SR-like proteins are associated with additional costs to cells that cancel out the decreased burden of the inefficiently spliced intron. See numerical data for this figure in S1 Data. WT, wild-type.

We next aimed to reveal which genetic changes may have occurred in *Trans* to the fusion gene. To that end, we fully sequenced the genomes of the two *Trans*-evolved colonies (A-*trans* and B-*trans*) and identified, respectively, three and two mutations in each of their genomes (see S2 Table for list of mutations). Interestingly, in both colonies we found a mutation in a member of the SR-protein group in *S*. *cerevisiae*, which are splicing-related genes. Specifically, in colony A-*trans* we found a nonsynonymous mutation in *GBP2*, changing histidine into tyrosine, H160Y, and in colony B-*trans* we found a nonsynonymous mutation in *NPL3*, changing phenylalanine into valine, F160V (Fig 7A).

Npl3 was previously shown to be loaded onto pre-mRNAs during early stages of transcription [48,49] and to support the recruitment of the spliceosome [29] after it has completed its quality control function to monitor correct 5′ capping (Schneider and Krebber, in preparation). Unlike for Npl3, deletion of Gbp2 does not result in accumulation of pre-mRNAs [29]. In contrast, Gbp2 was shown to associate with the late-stage spliceosome and appears to function as a quality control factor that oversees successful splicing of pre-mRNAs, which facilitates the recruitment of the nuclear exosome to incorrectly spliced pre-mRNAs in order to eliminate these faulty transcripts [33].

To examine whether these mutations in the two SR-like proteins are sufficient to increase fitness and YFP fluorescence of cells that harbor the low-efficiency spliced intron as part of the YFP-Kan construct, we used CRISPR technology (see Methods) to introduce these point mutations, each individually, to the background of the Splicing^Low^ ancestor. We termed these engineered strains *trans*-Rescue-A and *trans*-Rescue-B. Indeed, these two new rescue strains grew faster on the G418 antibiotics compared with ancestor cells (Fig 7B), and they both showed higher fluorescence intensity levels of YFP (Fig 7C). Hence, it appears that these *Trans* mutations result in increased levels of the YFP-Kan proteins.

Notably, both mutations we identified in *GBP2* and *NPL3* occurred in one of the two RNA recognition motifs of their encoded proteins (Fig 7A), suggesting that their affinity to the YFP-Kan transcript may have changed. We therefore tested if the mutant proteins would show modified YFP-Kan RNA-binding affinities compared with their wild counterparts. Mutant or wild-type (WT) Gbp2 and Npl3 were each immunoprecipitated with specific antibodies and the associated RNAs were purified (see Methods). Following reverse transcription, the levels of YFP-Kan mRNA bound to the different proteins were analyzed with RT-PCR. Interestingly, we obtained distinct results for Gbp2 and Npl3. While mutant gbp2 showed significantly decreased binding to YFP-Kan compared with WT, mutant npl3 seemed to have a slightly higher affinity to the reporter RNA (Fig 7D). These results are in agreement with our current understanding of the functions of the two proteins. The increase in YFP-Kan binding of the mutant npl3 might result in better assembly of the spliceosome and consequently increased splicing efficiency. The decreased binding of the mutant gbp2 to the YFP-Kan transcript might delay the assembly of the nuclear RNA degradation machinery and hence provide more time and higher chances to the YFP-Kan transcript to be successfully spliced and exported to the cytoplasm.

### *Cis* and *Trans* mutations show distinct phenotypes under permissive conditions

If the *Trans* mutations in the SR-like proteins are beneficial, why are they not the WT sequence? We speculated that these mutations are beneficial only under the drug pressure, and that they otherwise come with a cost when the antibiotic is absent. We thus measured the effects of these mutations on fitness in a medium lacking the antibiotics (rich YPD medium). We found that cells with either of the *Cis* mutations grew modestly faster than the Splicing^Low^ ancestor strain (Fig 7E), indicating that they alleviate some drug-independent burden of the inefficiently spliced intron in Splicing^Low^ cells. In contrast, cells with either of the *Trans* mutations grew similarly to Splicing^Low^ cells when the drug was not supplemented to the medium (Fig 7E). It seems then that the *Trans* mutations do not alleviate cellular fitness in the absence of the drug, i.e., they do not reduce the drug-independent burden of the inefficiently spliced intron.

## Discussion

In this work, we study the role of the splicing machinery in optimization of gene expression programs by placing selective pressure on cells to improve the splicing efficiency of a specific gene. Our results provide molecular evidence for the relevance of splicing as another instrument in the cellular toolbox towards adjusting its gene expression patterns. To the best of our knowledge, we demonstrate the first experimental evidence of splicing efficiency adaptation, confirming that this adaptation can occur in *Cis* and *Trans* similarly to adaptations of other stages of the gene expression regulation process. Adaptation of splicing efficiency might be very common among species evolution, given the observed correlation between splicing efficiency and transcription level of genes [28]. Hence, it is of interest that we elucidate more molecular mechanism that allow optimization of intron splicing.

Two potential solutions to the burden that we imposed on our Splicing^Low^ ancestor lines were surprisingly not realized during our lab-evolution. First, considering previous studies of splicing evolution, one could have expected the intron to be lost by a genomic deletion or through reverse transcription [50,51]. Such a solution could have been an ideal evolutionary adaptation to alleviate the burden, as we show that the intron-less strain has the highest fitness. The fact that we did not observe an intron-loss event suggests that this is a less accessible solution in this case, in agreement with previous evidence in yeast that nucleotide mutations are 33 times more frequent than deletion events [52]. It further seems that our evolving strains did not evolve a transcription- or translation-based solution that in principle could have elevated the expression of the YFP-Kan fusion gene. Instead, adaptation appears to have affected the splicing of the gene. Given that some of the adaptations were found within the intron, another surprise was that none of the mutations occurred within any of the intron’s three functional sites: the 5′ donor, 3′ acceptor, or the branch point of splicing. Indeed, one mutation that was verified here to affect splicing resides in a region of the intron not known to exert a major effect on splicing, and another splicing-improving mutation happened in the upstream exon. These observations indicate that various positions in the intron and its proximity may facilitate the splicing rate and take part in the evolution of introns, a phenomenon that was previously discussed only in regards to alternative splicing [53,54].

Interestingly, the *Cis* mutation that resides inside the intron results in considerable changes in the predicted RNA secondary structure of the intron, presumably lowering the association probability of the 5′ donor and branching point sites. This finding goes in line with previous observations that show how RNA structures can inhibit or facilitate binding of spliceosome components to the pre-mRNA and affect splicing efficiency [42,43], specifically at the edges of introns [36,42,43,55,56]. Thus, it is tempting to speculate that many more positions than previously recognized of introns’ sequences, rather than only its edges and the branching point, are under selection and are biologically relevant to the intron function.

Notably, the fluorescence intensity per protein molecule of the YFP domain was decreased due to the nonsynonymous mutation in the YFP first exon, suggesting that under certain evolutionary constraints, selection may hamper superfluous functions of certain protein domains so as to increase availability of the entire protein.

Adaptive changes also occurred in *Trans* to the YFP-Kan locus and increased availability of the splicing machinery to this gene. An alternative explanation for evolutionarily adaptive changes in splicing availability could be based on cellular physiological response. In such a model, the introduction of an inefficiently spliced intron into a gene that is on high demand may occupy a larger portion of splicing machinery, which in turn prevents splicing of other intron-containing genes, which ultimately leads to degradation at the pre-mRNA level, e.g., by the mRNA quality control machinery [33]. Although valid, this physiological adaptation appears less likely to explain our results. First, the physiological model predicts that the expression level changes that we see following the evolutionary adaptations will also be seen when comparing the expression of the splicing genes and intron-containing genes between Splicing^Low^ and the Control strain (that harbors no intron in its YFP-Kan construct). However, we did not observe such a difference, suggesting that a physiological regulatory model is less likely (Fig 6A). Second, the two *Cis*-rescue strains, which did not evolve and only harbor our artificially introduced *Cis*-acting mutation, did not demonstrate changes in splicing availability. Generally, evolved strains showed higher supply-to-demand rations for splicing compared with non-evolved strains in our study (Fig 6C). These observations more forcefully support an evolutionarily adaptive process that relies on additional genetic or epigenetic changes that are accumulated during the continuous growth of the evolved populations.

Recently, the competition of pre-mRNAs for the splicing machinery was shown to affect cellular function, as splicing efficiency of multiple introns was influenced by changes in the composition of the transcript pool [57]. While this mechanism was elegantly shown to take part in physiological adaptation by maintaining the separation between meiotic and vegetative gene-expression states, it is also possible that it can be used as an adaptive mechanism in evolution to optimize gene expression levels of cells.

Our findings demonstrate how availability of the splicing apparatus may have been adaptively increased both by elevating the expression level of the machinery’s genes and/or by reducing expression of other intron-containing genes that probably compete with the antibiotic resistance un-spliced RNA for the spliceosome. Thus, increase in supply-to-demand ratio, analogous to the case in translation systems [8,58], appears to have evolved in this case.

Additionally, we revealed a role for SR-like proteins, *GBP2* and *NPL3*, when the splicing machinery adapts to a new need of optimizing splicing of a specific intron. Notably, these proteins have various functions, including early recruitment of the spliceosome, quality surveillance of nascent mRNA quality, association with the nuclear RNA degradation machinery of faulty transcripts, and finally, assistance with nuclear export for mature mRNAs.

Interestingly, the nonsynonymous mutations we found in these proteins occurred in one of their RNA recognition motifs. Using an RNA immunoprecipitation assay, we showed that the mutations in Gbp2 and Npl3 decrease and increase, respectively, the binding capacities of the YFP-Kan construct. Yet, these surprising opposite effects can be explained by the different roles of Gbp2 and Npl3—the former is a quality control factor of splicing that elicits degradation of un- or mis-spliced transcripts, and the latter is a spliceosome-recruitment agent. On the one hand, the lower binding capacity of the mutated Gbp2 probably provides more time for the spliceosome to complete the maturation of the YFP-Kan pre-mRNA before it is degraded. The improved binding capacity of the mutated Npl3, on the other hand, facilitates the recruitment of the spliceosome and hence might improve splicing efficiency of the transcript. The *Trans*-evolution mechanism we reveal here is intriguing because it shows that under acute selection, a cellular machinery can evolve for the need of one gene only.

Ultimately, we revealed a fundamental difference for *Cis* and *Trans* evolution of the splicing machinery when this cellular process faces a need to adapt. While *Cis*-based adaptations are “local” and lowered the burdens of splicing for the intron under selection, *Trans*-based adaptations showed wider cellular effects that may be costly to cells when the original evolutionary challenge is lifted. Further investigations will reveal which of these solutions, *Cis* or *Trans*, proves to be more evolutionarily stable—to fully reveal the dynamics of splicing adaptation when cells optimize their gene expression.

## Methods

### Yeast strains and plasmids

All *S*. *cerevisiae* strains in this study have the following genetic background: his3Δ1::TEF2-mCherry::URA3::RPS28Ap-YiFP-KAN::NAT; canΔ1::STE2pr-Sp_his5; lypΔ1::STE3pr-LEU2; leu2Δ0; ura3Δ0.

Strains of Y-intron-FP were taken from Yofe and colleagues [36] and were introduced with a Kan resistance gene fused 3′ terminally to the YFP. To reconstitute the mutations discovered after lab evolution (rescue strains), we amplified cassettes of Y-i_mut_-FP-KAN and transformed these into the ancestor Control strain, selecting with KAN. Notably, all strains also carry an mCherry-fluorescent protein driven by an independent *TEF2* promoter that was used to normalize cell-to-cell variability for the YFP-Kan expression levels.

### Media

Cultures were grown at 30°C in rich medium (1% bacto-yeast extract, 2% bacto-peptone, and 2% dextrose [YPD]). Throughout all experiments, G418 was supplemented to the medium at a concentration of 3 mg/mL, which is 10-fold higher than the standard.

### Evolution experiments

Lab-evolution experiments were carried out by daily serial dilution for 80 days. Cells were grown on 1.2 mL of YPD+G418 at 30°C until reaching stationary phase and then diluted by a factor of 1:120 into fresh media (approximately 7 generations per dilution, a total of about 560 generations).

### Liquid growth measurements

Cells were grown in YPD+G418 at 30°C overnight. The following day, they were diluted to an OD = 0.05 in YPD+G418, and optical density (600 nm) measurements were taken at 30-minute intervals. Growth comparisons were performed using 96-well plates, and the growth curve for each strain was obtained by averaging at least 15 wells.

### Flow cytometry measurements of YFP-Kan levels

Cells were grown in YPD+G418 at 30°C overnight. The following day, they were diluted to an OD = 0.05 in YPD+G418, placed at 30°C, and followed until they reached logarithmic growth phase at an optical density of between 0.4 and 0.5. Then, YFP and mCherry levels were measured for between 20,000 and 50,000 cells for each culture with flow cytometry. Gating was performed according to side and forward scatters, and YFP levels were normalized with the mCherry signal for each cell individually.

### Quantitative PCR measurements of splicing efficiency

Cultures were grown in YPD+G418 at 30°C until cells reached the logarithmic growth phase at an optical density of approximately 0.4. Then, RNA was extracted using MasterPure kit (Epicentre) and were reverse transcribed to cDNA using random primers. A total of 2 μL of cDNA was added to each reaction as template for qPCR using light cycler 480 SYBR I master kit and the LightCycler 480 system (Roche Applied Science), according to the manufacturer’s instructions. For each strain, qPCRs were performed with two to three biological repetitions and three technical repeats. A first qPCR was performed targeting the transcript-spliced version, with a forward primer complementing the exon-exon junction and a downstream reverse primer. A second PCR targeted the un-spliced version of the transcript, with a forward primer complementing the intron and the same reverse primer of the first reaction: F_exon-exon_ = 5′-CACTACTTTAGGTTATGGTTT-3′; F_intron_ = 5′-CTTCAATTTACTGAATTTGTATG-3′; R_both_ = 5′-GTCTTGTAGTTACCGTCA-3′.

Splicing efficiency is reported as the average Cp of the spliced transcript minus the average Cp of the un-spliced version.

### mRNA deep sequencing

Cultures were grown in YPD+G418 at 30°C until cells reached the logarithmic growth phase at an optical density of approximately 0.4. Cells were then harvested by centrifugation and flash-frozen in liquid nitrogen. RNA was extracted using a modified protocol of nucleospin 96 RNA kit (Machery-Nagel). Specifically, cell lysis was done in a 96-deep-well plate by adding to each well 450 μL of lysis buffer containing 1 M sorbitol, 100 mM EDTA, and 0.45 μL lyticase (10 IU/μL). The plate was incubated at 30°C for 30 minutes to break cell walls and centrifuged for 10 minutes at 3,000 rpm, followed by the removal of the supernatant. Then, extraction continued as in the protocol of nucleospin 96 RNA kit, only using β-mercaptoethanol instead of DTT. Poly(A)-selected RNA extracts of approximate size of 200 bps were reverse transcribed to cDNA using poly(T) primers that were bar coded with a unique molecular identifier (UMI). cDNA was then amplified and sequenced with an Illumina HiSeq 2500.

### Analysis of mRNA deep sequencing

Processing of RNA-seq data was performed as described in Voichek and colleagues [59]. Shortly, reads were aligned using Bowtie [60] (parameters:—best–a–m 2 –strata -5 10) to the genome of *S*. *Cerevisiae* (R64 from SGD) with an additional chromosome containing the sequence of the YFP-Kan construct. For each sequence, we normalized for PCR bias using UMIs, as described in Kivioja and colleagues [61]. Next, reads for each gene end (400 bp upstream to 200 bp downstream of the ORF’s 3′ end) were summed up to estimate the gene’s expression level. Genes with coverage lower than 10 reads were excluded. To normalize for differences in coverage among samples, we divided each gene expression by the total read count of each sample and then multiplied by 10^6^. Then, the expression ratio was calculated between an evolved/rescue colony to the ancestor, and a log_2_ operation was performed on that ratio. These values were used to compare expression levels of gene groups (ribosomal genes, general stress response genes, splicing machinery genes, intron-containing genes) and of the YFP-Kan mRNA levels as described in the manuscript. When calculating the expression levels of splicing machinery and intron-containing gene groups, the ribosomal and general stress response genes were excluded from the analysis in order to avoid bias from cellular regulation due to changes in physiology and growth rate of the cells.

### CRISPR genome engineering

CRISPR protocol for *S*. *cerevisiae* was performed as described previously [62]. Shortly, gRNA sequences targeting a locus near (<50 bps) the position of the desired mutations were cloned into the vector bRA89 that allows co-expression of Cas9 and gRNA. CRISPR plasmid (1 μg) was co-transformed to yeast cells (20 μL) along with a repair dsDNA cassette with the desired mutations (500 ng) using the standard yeast transformation protocol. Cells were plated on selective plates (YPD+hygromycin, 50 mg/mL), colonies were screened with PCR and Sanger sequencing for the desired mutations, positive colonies were grown on permissive media for 24 hours to ascertain loss of the CRISPR plasmid, and copies were stored at −80°C.

The repair cassette holds 45-bp homology on each end, flanking the desired mutation. Because the mutation did not modify the PAM sequence, additional synonymous mutations were also introduced alongside the desired mutation to stop the cutting of the locus by the Cas9+gRNA complex. These synonymous mutations were also introduced to the relevant genetic background without the desired mutations and were confirmed to have no effect on cellular growth or YFP expression levels.

### RNA co-immunoprecipitation

Yeast cells were grown in YPD at 25°C until logarithmic phase and collected via centrifugation. Cells were resuspended in 1× pellet volume RNA-IP buffer (25 mM Tris-HCl [pH 7.5], 150 mM NaCl, 2 mM MgCl_2_, 0.5% [v/v] Triton X-100, 0.2 mM PMSF in isopropanol, 0.5 mM DTT, 40 units of RiboLock RNase Inhibitor [Thermo Fisher Scientific], cOmplete EDTA-free Protease Inhibitor Cocktail [Roche Diagnostics and Sigma-Aldrich]) and lysed with vigorous vortexing three times, 20 s, 6 m/s with the FastPrep-24 Instrument (MP Biomedicals) in the presence of 1× pellet volume of glass beads. For immunoprecipitation, cleared lysates were incubated with or without Gbp2 or Npl3 antibody (rabbit polyclonal, self-made) at 4°C for 1 hour followed by the addition of 10 μL Protein G sepharose beads (Amersham Biosciences) and further incubation at 4°C for 2 hours. RNase-Free DNase I (QIAGEN) was also added to digest DNA during incubation. A total of 100 μL of the lysate was treated with DNase I and incubated at 4°C in parallel. The beads were washed five times with RNA-IP buffer. RNA was isolated from both lysate and eluate samples using the TRIzol Reagent (Thermo Fisher Scientific) and eluted in 20 μL DEPC-treated ddH_2_O. Prior to reverse transcription, the RNA samples were treated with TURBO DNA-*free* Kit (Thermo Fisher Scientific) to eliminate residual DNA.

### Reverse transcription and qRT-PCR of precipitated RNA

The same concentration of RNA from all samples was reverse transcribed using the FastGene Scriptase II cDNA Kit (NIPPON Genetics EUROPE) with Random Hexamer Primer (Thermo Fisher Scientific). PCR samples were prepared with qPCRBIO Sygreen Mix Lo-ROX (PCR Biosystems), and qRT-PCR was performed using the CFX Connect Real-Time PCR Detection System (Bio-Rad Laboratories) for 45 cycles with an annealing temperature of 60°C. Forward primer (5′-TTATTCACTGGTGTTGTCCC-3′) and reverse primer (5′-CATGGAACTGGCAATTTACC-3′) that bind specifically to *YFP-Kan* were used. Each PCR reaction was performed in triplicates, and the average Cq value was used in further analysis. From each eluate Cq value, the corresponding lysate Cq value was substracted (ΔCq). The ΔCq of the no-antibody control sample was subtracted from the ΔCq of the pull-down sample (ΔΔCq). Binding of the RNA was calculated by 2^−ΔΔCq^.

## Supporting information

S1 Fig Supply-to-demand ratio in each of the evolved colonies. Folding change ratios in log_2_ are shown for splicing genes (left) and intron-containing genes (right). Black line represents the median of the distribution. See numerical data for this figure in S1 Data.
(PDF)

S1 Data Supporting data for all main figures. (XLSX)

S1 Table List of strains used in this work. (XLSX)

S2 Table List of genomic mutations in *Trans*-evolved colonies. (XLSX)

## Abbreviations

kan: kanamycin resistance gene
UMI: unique molecular identifier
WT: wild-type
