# Supplementary figures and images for: Evolution of intron splicing towards optimized gene expression is based on various Cis- and Trans-molecular mechanisms
Source: PLoS Biol. 2019 Aug 23;17(8):e3000423. doi: 10.1371/journal.pbio.3000423 (PMC6728054; doi:10.1371/journal.pbio.3000423)

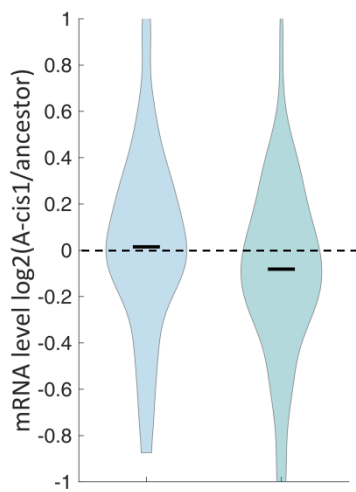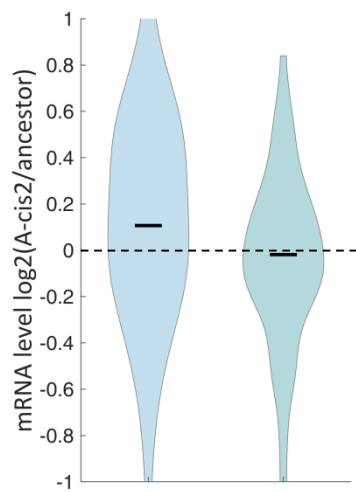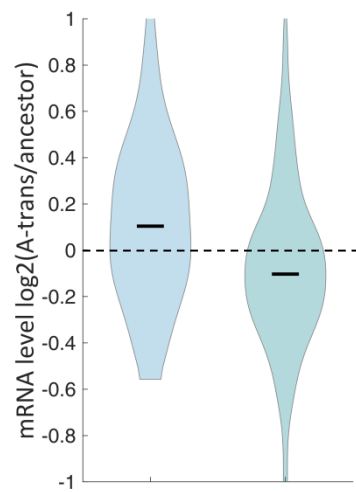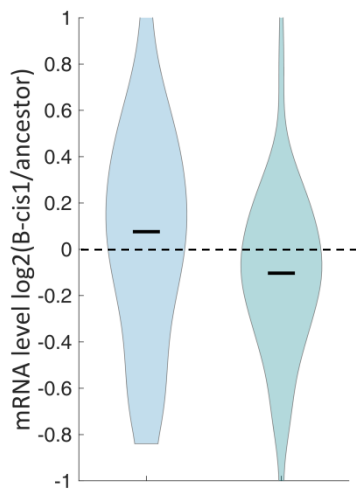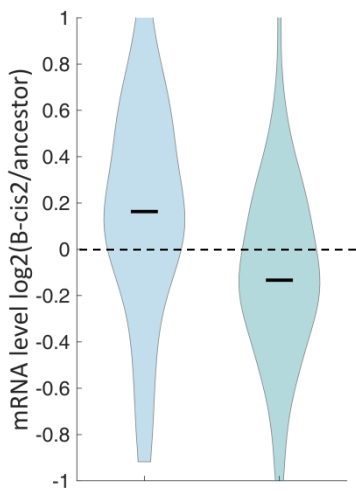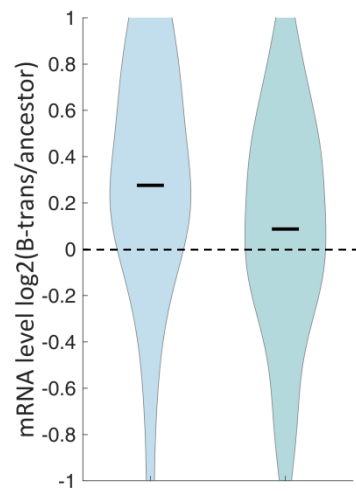

Supplement: S1 Fig — Folding change ratios in log2 are shown for splicing genes (left) and intron-containing genes (right). Black line represents the median of the distribution. See numerical data for this figure in S1 Data. (PDF) [file pbio.3000423.s001.pdf]
